# Damage Kinetics at the Sub-micrometric Scale in Bast Fibers Using Finite Element Simulation and High-Resolution X-Ray Micro-Tomography

**DOI:** 10.3389/fpls.2019.00194

**Published:** 2019-02-21

**Authors:** Sofiane Guessasma, Johnny Beaugrand

**Affiliations:** ^1^INRA, UR1268 Biopolymères Interactions Assemblages, Nantes, France; ^2^INRA, URCA, UMR614, Fractionnement des AgroRessources et Environnement, Reims, France

**Keywords:** bast fiber, X-ray micro-tomography, tensile properties, damage kinetics, finite element simulation, microstructure

## Abstract

This study combines experimental testing and computation analysis to reveal the role of defects and sub-micrometric microstructure in tensile behavior of hemp bast fibers. In particular, these structural defects represent the footprint of the processes to which the fibers elements are subject along the whole transformation chain from the plant to the end use product. Tensile experiments performed on elementary fibers and bundles in a wide diameter range (40–200 μm) are simultaneously conducted with X-ray micro-tomography observation. 3D images of ultra-fine resolution (voxel size of 280 nm) are achieved at different deformation magnitudes up to the complete failure thanks to the use of synchrotron radiation (ESRF, Grenoble, France). A Finite element (FE) model is implemented based on the conversion of the tomograms into 3D meshes. High performance computing is used to simulate the tensile response of the hemp bast fibers. In particular, the effects of notching and sub-micrometric structure of the fibers are explored. Results show the presence of different types of diffuse damage kinetics, which are related to the variability in the fiber size, surface defects and the presence of the lumen space. The damage behavior is found to be sensitive to the type of stress criterion implemented in the FE computation. The predictive analysis demonstrates the relevance of using embedded microstructure simulations to reveal the extent of stress localization and predict the failure properties in bast fibers for innovative composite manufacturing for instance.

## Introduction

Fiber demand continues to increase despite the recent Financial Crisis of 2008, when the world economy faced perhaps its most dangerous crisis since the great depression of 1928, according to economists. Both the synthetic and the cellulosic fiber production trends are linearly indexed on the gross domestic product. Following the source data from Tecnon OrbiChem company, the future of cellulosic fibers is promising more particularly because of positive projections driven by the Asian countries production trend (Qin, 2014). With regards to global market demand of natural fibers compared to the synthetic market share, the situation evolved significantly toward natural fiber consumption encouraged by initiatives such as the International Year of Natural Fibers hold in 2009. According to the cotton promotion international forum, natural fibers continue to grab market share in textile industry and the overall consumption crosses the level of 20 million tons in 2005. This is half of the chemical consumption for the same end-use. In plastics industry (Mohanty et al., 2018) and more particularly in automotive industry (Carus and Eder, 2014), the situation is more contrasted, i.e., no volume increase between 2012 and 2015 was monitored in EU when the natural fiber total production and applications amounts continue to rise steadily (Carus and Partanen, 2018). The potential of natural reinforced plastics as light-weight structures sharing most of the technological processing routes with fossil-based composites is now proved (Müssig, 2010; Summerscales et al., 2010; Shah, 2014). In 2012, European automotive industry demand represents 92,000 tons among which hemp usage represents 4000 tons (Carus et al., 2014). It is expected that such demand increases by a factor of 5 if stimulated by the accompanied initiatives and policy support. More recent reports support partially this expectation at the worldwide level (Food and Agricultural Organization of the United Nations [FAO], 2015).

If the market progress depends on trade volumes and raw material price index, this logic induces some side effects like the fact that all biosourced composites are not all eco-friendly. A typical example is the use of exotic fibers that are imported from distant regions in the manufacturing process of bio-based composites (Dissanayake et al., 2009; Le Duigou and Baley, 2014).

In the specific case of hemp, the environmental footprint tips the scales in favor of bio-based composites because of the energy and greenhouse gas release savings in comparison with fossil-based materials (La Rosa et al., 2014). This is evidenced by life cycle assessment, which promotes the development of hemp-based composites (Le Duigou and Baley, 2014). Indeed, hemp, as an annual plant, stores more than 1 CO_2_ kilogram per kilogram of hemp. Regarding the carbon storage, values of 30 to 75% saving are reported compared to synthetic fibers. In addition, energy cost for hemp production is equivalent to six times the cost production for glass fibers.

In Europe and according to the European industrial hemp association, this economic sector suffers from the lack of regulation, lower production trends and competition with other end-uses such as biofuel sector (Carus et al., 2014). As an industrial part, hemp fiber makes the difference as reinforcement only if environmental costs and specific performance are accounted. The direct support from the political decision makers to the greening framework requires proper choices to be made starting from the plant to the transformed material. The end-use criteria play the most significant role in triggering the whole chain. This is precisely what is not well-known for hemp-based parts for various reasons. Some of these reasons related to the raw material itself are exposed hereafter for the case of hemp.

In many structural engineering situations, strength and stiffness are important ranking criteria for fiber selection (Bourmaud et al., 2018). Hemp as a natural fiber shares the common drawback of varied quality, which limits the predictability of its mechanical performance. Inhomogeneity of the lignocellulosic fibers (Li et al., 2013; Bourmaud et al., 2017) and their variations in terms of quantity and quality are still under investigation. Such variability is still hardly predictable, even though it is now established that it originates from botanical and tissues origins, growing conditions (Müssig and Amaducci, 2018), harvesting date and first transformation like the decortications processing (Müssig, 2010; Placet et al., 2017). Hemp belongs to the bast fiber, and as so, hemp fibers faced some shortcomings in terms of chemical compositions, structural impacts on moisture absorption, and consequently on the mechanical properties. The hydrophilic nature of the hemp fiber is generally detrimental for biocomposites (Dhakal et al., 2007, 2014) but recent development on hygromorph biocomposites (Le Duigou et al., 2019; Péron et al., 2019) highlight promising development based on this hydrophilic characteristic. From the structural viewpoint, the presence of multiple flaws combined to the large surface-to-volume ratio contributes significantly to increase the scatter of mechanical parameters reported in the literature (Fan, 2010). For instance, recent studies on hemp show Young’s modulus in the range 15–45 GPa, and tensile strength ranging from 280 to 1700 MPa (Marrot et al., 2013). The mechanical performance dependence on fiber dimensions (Thomason and Carruthers, 2012; Placet et al., 2014) and accuracy of the measurement (Legland and Beaugrand, 2013; Hamdi et al., 2015; Garat et al., 2018) are serious concerns for attempting a proper evaluation of the technical part performance against failure.

Defects such as external fibrillation can weaken the hemp fiber structure (Pickering et al., 2007), which makes the fracture of the material highly defect sensitive. Therefore, there is a need to achieve a relationship between the fracture performance and the defect size for different hemp fiber dimensions. In order to achieve a better understanding of the role of surface flaws on rupture properties of hemp fibers, this study follows a strategy that combines 3D imaging of the fiber microstructure, experimental testing and numerical analysis based on finite element (FE) computation. Starting from bundles or individualized fibers, laser ablation process is performed on hemp specimens to create notches of different sizes and geometries (U- and V-notched). These notches act as effective defect sites to concentrate stress during loading and trigger rupture of the natural fiber. Micromechanical testing under tension loading conditions is then conducted for varieties of conditions. Tensile experiments are simultaneously conducted with x-ray micro-tomography imaging using ESRF facility at Grenoble, France. A FE model is developed, as a second step, based on implementation of the real 3D microstructure of the hemp bast fibers. The model uses also the experimental loading conditions and the fiber material properties with the main hypothesis of homogeneous properties for the solid phase combined to an explicit implementation of the void structure.

## Materials and Methods

The hemp fibers (*Cannabis sativa* L, variety Fedora 17) are supplied by Fibers Recherche Développement^®^ (Troyes, France). Bundles and elementary fibers are manually selected based on stereomicroscopy quality control. Notches are performed using PALM^®^ MB IV laser micro-dissector from ZEISS, which uses a high-energy laser beam directed by high-precision optics. The laser operates at a wavelength of 355 nm, a working frequency of 100 Hz and beam energy of 90 μJ. Visualization of the ablation process is done through a ZEISS AXIO OBSERVER Z1 (Carl Zeiss, Oberkochen, Germany) inverted microscope.

**FIGURE 1:**
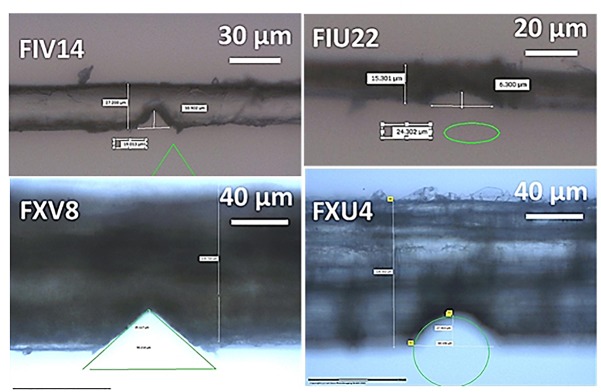
Optical images illustrating four tested hemp fibers: (left top) V-notched elementary, (left bottom) V-notched bundle, (right top) U-notched elementary, and (right bottom) U-notched bundle. Notching is achieved using laser micro-dissection and the geometrical shape (triangle or ellipse) expected are superimposed on the micrograph in green.

Notches are controlled with an accuracy of 1 μm. PALM robosoftware solution is used to guide the ablation process and notch design of different sizes and geometries. Selection of the region of interest from the fiber is done with different magnifications (20 and 40×) depending on fiber dimensions. All notches are performed under 40× magnification which is convenient to all attempted depths (2 and 30) μm. Two notch geometries are considered, namely V- or U-type (Figure 1). Notch openings (w), depths (h) and fiber diameters (d) are the main control parameters. A scaling quantity between w and d is introduced as the opening angle using

(1) tan(θ/2)=w/(2×h)

where θ is varied between 26 and 127 degrees thanks to a large selection of notch widths 4–108 μm.

Hemp bundles and elementary fibers correspond to a broader choice of d, which evolves between 13 and 224 μm. This range is coupled to large scaled notch depth (h/d) in the range (0.1, 0.6). The size of the notch is bounded naturally from the lower bound by the laser resolution and from the upper bound by the lateral dimension of the fiber. If the notch size is too small, there is a high risk to whiteness the crack departure from the surface defects rather than from the notch itself. If it is too large, there will be not enough frames to capture the damage growth. Thus, the interval selected in this study satisfies both constrains.

**FIGURE 2:**
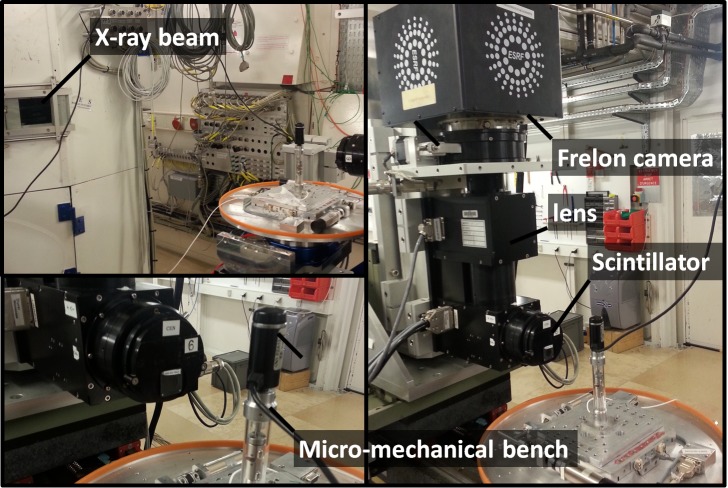
Experimental setup at synchrotron radiation facility at ESRF Grenoble, France showing the tensile testing equipment and the X-ray micro-tomography imaging system.

Micromechanical testing is performed on notched hemp specimens of 20 mm in length using a test bench from 3SR laboratory, Grenoble, France (Figure 2) mounted on a rotating holder. The tensile experiments are conducted up to the failure of the fiber with a load cell of 50 N capacity and a displacement speed of 1.25 μm/s. Testing of the notched fibers is conducted at room temperature and under a relative humidity of 50%. Loading is transmitted to the fiber via a flat sheet according to a well-known fiber testing configuration, which avoids pre-tension of the fiber and ensures its alignment during loading. The tested specimen is glued using high-viscosity epoxy droplets (Araldite bi components from Bostick S.A.) on a stiff cardboard of grade 160 g/m^2^. The center part of the sheet is removed using press-cutter equipment (SAS NONAIN, Advanced Mechanical Tools, Saint-Brice-Courcelles, France). The gage window has a length of approximately 2 mm ± 5%. Load transfer to the specimen is allowed by cutting strips connecting the gage window to the grips using pedicure scissors. So, the boundary conditions on each end of the fiber is as follows. One end is constrained against displacement in all space directions. The other end is only allowed to move in the loading direction, which aligned with the fiber longitudinal direction.

The test configuration is adapted to allow simultaneous 3D imaging on the beam line ID19 from ESRF, Grenoble, France. The beam energy is adjusted to 19 keV and 1400 projections are needed to obtain the tomograms of deformed fibers. These radiographic images are obtained by a Frelon camera (2048 × 2048 pixels) developed at ESRF coupled to GGG scintillator (thickness 10 μm) and an optical × 20/NA 0.45 objective from Olympus. Images are obtained at four different load levels up to the fiber rupture. The resolution of each image is 8.6 × 10^9^ voxels, with a voxel size of 280 nm. This corresponds to a field of view of 573 μm^3^. Conversion of the 2D radiographic images into 3D tomograms is achieved using filter-back projection algorithm. Processing of the 3D images is conducted using the public domain ImageJ software. Four different configurations are studied including hemp bundles and elementary fibers with V-like and U-like notches. The force-displacement responses are collected for each combination.

### Finite Element Computation

**FIGURE 3:**
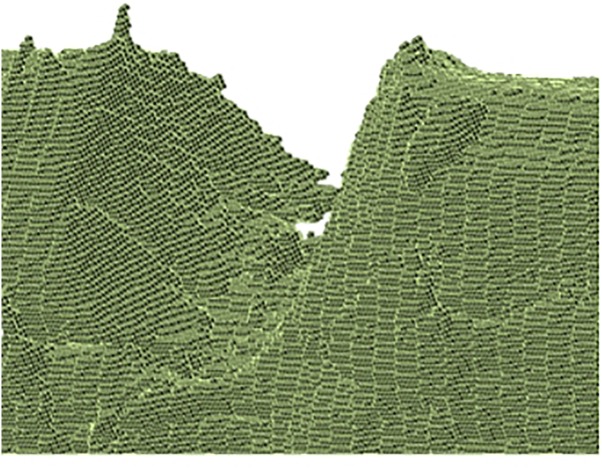
Magnified view close to a U-notch on digitalised hemp fiber showing 3D regular meshing using voxel-to-element conversion.

Finite element computation is used to predict the tensile behavior of notched hemp fibers based on 3D image implementation. Only the 3D images of the initial load steps are used for the implementation of the FE model. The other load steps are used for qualitative and quantitative evaluation of the damage growth in the hemp bast fiber. This scheme considers a conversion of voxels into cuboid elements with each element described by eight nodes (Figure 3). Each node has three degrees of freedom corresponding each to the translations UX, UY, and UZ in the space direction X, Y, Z.

The fiber geometry is built without reduction in the image resolution, where the model size varies from 61 × 10^6^ to 269 × 10^6^ dof, where one dof refers to the displacement degree of freedom. ANSYS mechanical software (ANSYS, Inc., Canonsburg, PA, United States) is used as a framework for all computations. A linear elasticity scheme is associated to the meshed structure. This scheme is able to represent truly the observed force – displacement response of the notched fibers. Elastic and isotropic material properties are associated to the meshed part, namely Young’s modulus E(x,y,z) and Poisson’s coefficient V (x,y,z). All these quantities are spatially dependent on the gray level g_xyz_ of the fiber elements representing the sub-structure of the fiber. After segmentation, the two remaining gray levels (0 and 255) correspond to the air and the solid phases. The following relationships are considered to spatially assign the solid and air properties depending on the local gray level. Thanks to the use of the Kronecker function (δ), which selects one of the two main gray levels, the mesoscopic heterogeneity of the fiber is implemented.

(2) $$\text{E}(\text{x},\text{ y},\text{ z})={\text{E}}_{0}\times \text{δ}[1-(255-{\text{g}}_{\text{xyz}})/255]$$

and

(3) $$v(\text{x},\text{ y},\text{ z})=v_{0}\times \text{δ}[1-(255-{\text{g}}_{\text{xyz}})/255]$$

Constant engineering parameters (E_0_, v_0_) are used for the solid phase. All these parameters are adjusted to match the observed behavior of the fibers. Basically there is no need to use a complex identification procedure to obtain (E_0_, *v*_0_). The slope of the experimental curve is only dependent on one parameter, namely Young’s modulus. The identification procedure assumes an initial guess and a correction based on a linear function is enough to determine the true modulus that fits the experimental slope. This process has to be repeated for each fiber because the pore distribution and amount differ from one fiber to another. Poisson’s ratio can only be approached from the second load step, where the average lateral contraction is also compared to the one obtained from the deformed images. The same linearity is assumed to derive v_0_.

We use the experimental conditions to derive force – displacement response, which are compared to the observed ones taking into accounts four combinations: (V-notched, U-notched) × (fiber, bundle). The numerical boundary conditions mimic the experimental conditions. These correspond to a fully constrained end against displacement in all space directions. This also means that all degrees of freedom (U_x_, U_y_,U_z_) are grounded. The nodes belonging to the other end are only allowed to move in the Z direction (U_x_ > 0), while the remaining degrees of freedom are fixed (U_y_ = U_z_ = 0). The following relations between the degrees of freedom (U_x_,U_y_,U_z_) are implemented, accordingly:

(4) $${\left({\text{U}}_{\text{x}},{\text{ U}}_{\text{y}},{\text{ U}}_{\text{z}}\right)}_{\text{x}=0}=(0,\;0,\;0);\;{\left({\text{U}}_{\text{x}},{\text{ U}}_{\text{y}},{\text{ U}}_{\text{z}}\right)}_{\text{x}=\text{L}}=(\text{U},\;0,\;0)$$

where L is the length of the hemp fiber element and also represents the dimension in the loading direction.

The present linear elastic problem is solved in 3D to capture the spatial distribution of the stress in the fiber element and particularly the evolution of the stress state up to the rupture stage. In order to capture the damage kinetics, various damage criteria are implemented. All these criteria depend on the maximum stress levels selected among the quantities: stress intensity S_I_, principal stress component S_1_, and orthogonal normal stress components S_XX_, S_Y Y_, S_ZZ_, and shear stress components S_XY_, S_XZ_, S_Y Z_. At each load point, the properties of the elements of the highest stress levels converted into the ground level according to the scheme reported in an earlier study (Khelifa and Guessasma, 2013). Damage accumulation and the stress distribution are monitored as a function of the load increment. Because of the iterative process requiring multiple quasi-static solutions to be derived, the typical computation time per single load increment varies between 7 and 28 min. The damage analysis of the different bast fibers requires a total of 2,619 FE runs and a total of 38,466 min of CPU time.

## Results and Discussion

### Microstructure of Hemp Bast Fibers

**FIGURE 4:**
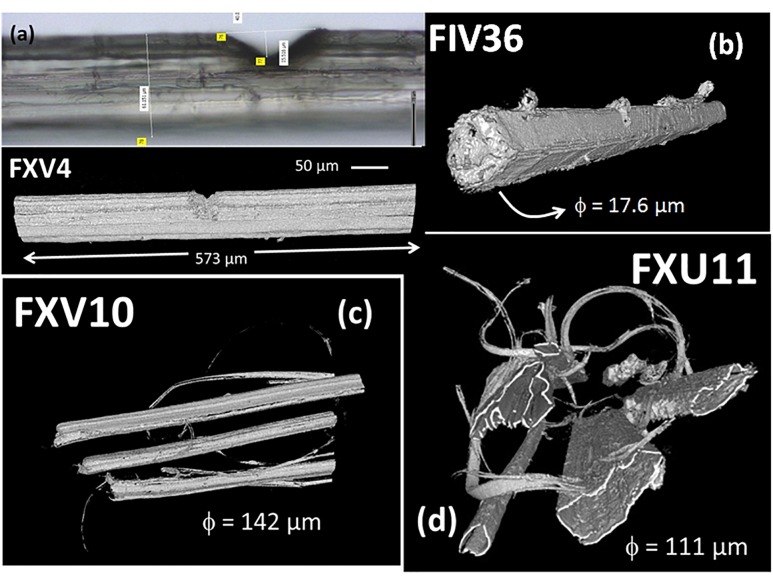
X-ray micro-tomography and corresponding optical images of hemp bundles and elementary fibers under various conditions: **(a)** bundle with V-notch acquired in a typical volume of 456 × 465 × 2048 voxels; **(b)** elementary fiber with a V-notch (typical volume 798 × 340 × 1846 voxels). Multiple elementary fibers in several bundles acquired in typical volumes **(c)** 1389 × 1722 × 1756 voxels and **(d)** 1004 × 970 × 2048 voxels. The voxel size is 280 nm in each case.

Figure 4 shows typical hemp bundle and elementary fibers imaged using X-ray micro-tomography. These images are processed to isolate the fibrous structure from the background using series of dilation and erosion operations and gray level thresholding. The bundle structure in Figure 4a has a diameter of 61 μm with a V-notch covering a depth of 15 μm and a width of 40 μm. This bundle exhibits a significant amount of surface flaws and an ellipsoid like cross-section form. As a contrast to the bundles, the elementary fiber shown in Figure 4b has more rounded cross-section form with typical dimension of 14 μm and a slight V-notch of 2.8 μm in depth and 7.7 μm in width. Despite the relative smooth shape of the elementary fiber, there some surface defects that still exist. The examination of the fibrous arrangements in Figure 4c,d reveals a large discrepancy in lateral dimensions, varied forms of cross-section areas, which typically call for the footprint of the defibrillation process.

**FIGURE 5:**
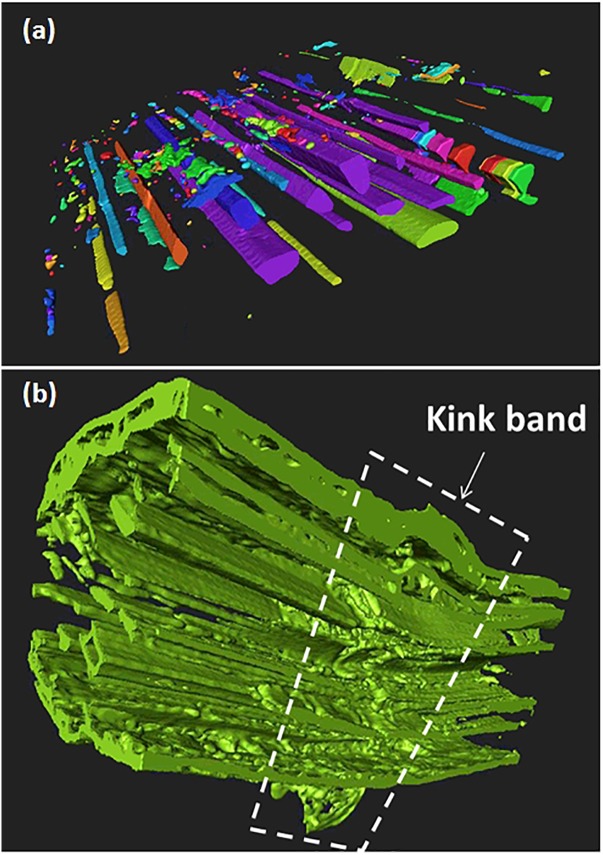
3D imaging of defects illustrated in hemp bast fibers: **(a)** tubular lumen space revealed using corner flooding, and 3D labeling technique on bundle FXU5, **(b)** longitudinal cross-section view near a collection of kink bands along the radial dimension of the bundle (white frame).

**FIGURE 6:**
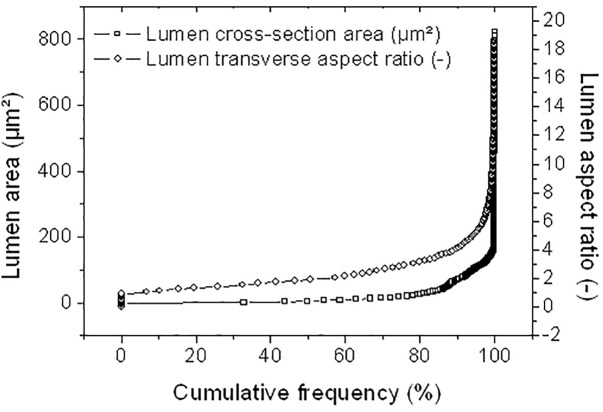
Statistical analysis on lumen structural attributes (cross-section area and aspect ratio) in the transverse dimensions.

With nearly 10% of porosity content measured in this study, the hemp bast fibers have a low porosity but this porosity has the drawback to expand over the entire length of the fiber. The tubular nature of the porosity, referred here as the lumen space, is a major structural feature of the bast fibers. Statistical analysis conducted on all cross-section views in the stack shown in Figure 5a determines the range of variation of both the lumen cross-section area and aspect ratio assuming ellipse-like approximation of the lumen transverse dimensions. The results illustrated in Figure 6 reveal that the average area of the lumen space is about 21 μm^2^ and a median value of only 4.9 μm^2^. The lumen has an elliptical shape with an average transverse shape factor (defined as the ratio between the major and minor semi-lengths) of 2.43 and a median value of 1.95.

The kink bands that are imaged in Figure 5 are other type of defects originated from the fiber, arguably generated during the extraction process. Together with the surface flaws, these defects trigger the mechanical performance of the bast fibers as revealed by the *in situ* experiments.

### Predicted Damage Kinetics

**FIGURE 7:**
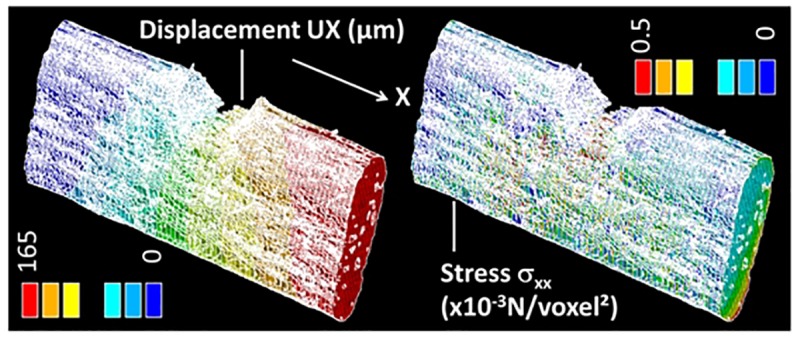
Finite element (FE) computation results showing the displacement component UX and stress component σ_XX_ for a typical tensile loading by 8% with respect to the original length in X-direction of a hemp bundle with a U-notch (FXU4).

Figure 7 depicts typical FE results related to the tensile loading simulation of a hemp bundle with U-notch (FXU4). This bundle represents the assembly of a large number of elementary fibers with the largest lumen space (porosity of 9%) among the imaged hemp bast fibers. It has also the most elongated cross-section surface with a shape factor of 3.23 and an average lateral dimension of 232 μm. The computation is performed on a representative volume containing 0.32 billons voxels (6.97 × 10^6^ μm^3^) for a typical length of 573 μm.

The separation between the solid elements within this volume is not possible. For instance, the properties of the middle lamellae are not separated from the rest of the technical fiber. This is because the model is based on the separation between the air and solid phase as suggested in Eqs (1) and (2). Looking into the differences between the solid phase properties means that there is enough contrast between the material elements to allow the segmentation of the different parts of the fiber. This is not directly accessible from the X-ray micro tomography imaging and would suggest, for instance, the coupling of this technique with X-ray diffraction.

The predicted displacement field UX along the loading direction is coherent with the implemented boundary conditions. The gradient of the displacement is, however, modified near the notch due to the localization occurring when the fiber is loaded. The predicted displacement field could be compared with the *in situ* synchrotron tomograms using for instance 3D strain mapping as suggested in Rjafiallah and Guessasma (2011) and Moussawi et al. (2014). However, the large time step associate with the 3D image acquisitions do not allow the deformation field to be captured. This is because of the large amount of discontinuities that appear during the loading stage, which are related to the damage development.

**FIGURE 8:**
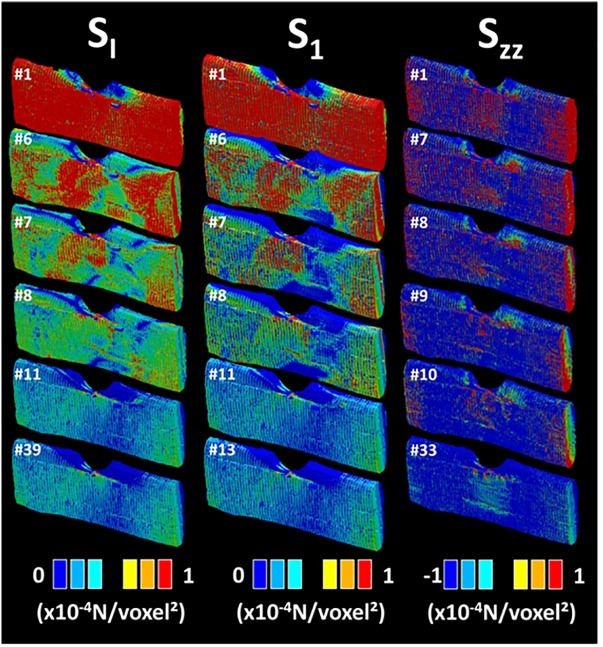
Finite element computation results showing the stress distribution evolution as a function of the load level for different damage criteria: S_I_, S_1_, and σ_ZZ_ (U-notch bundle FXU4). The load level label is indicated for each sequence.

The consequence of the stretching on the stress component σ_XX_ is the development of a heterogeneous field with areas of stress concentration near the notch and close to the loading ends. In addition, the presence of a surface texture allows varied stress magnitudes on the surface of the fiber. The nodal results shown in Figure 7 correspond to the first FE run prior damage initiation. In Figure 8, the full sequence of damage simulation is illustrated for three stress criteria, namely stress intensity S_I_, principal stress component S_1_, and normal stress component S_ZZ_. The decrease of the stress levels as a function of the load increment attests for the development of localized damage in the bundle. The nature and extent of the damage is fully dependent on the type of the damage criterion. Significant drop in the stress magnitude is achieved when the stress intensity or the principal stress criteria are used. For these two criteria, the regions where the stress decreases first are close to the notch and in a lesser extent at different locations at the surface of the bundle. A particular highlighted feature is that damage tends to connect regions of higher stresses, for instance, the bundle ends with the region near the notch.

**FIGURE 9:**
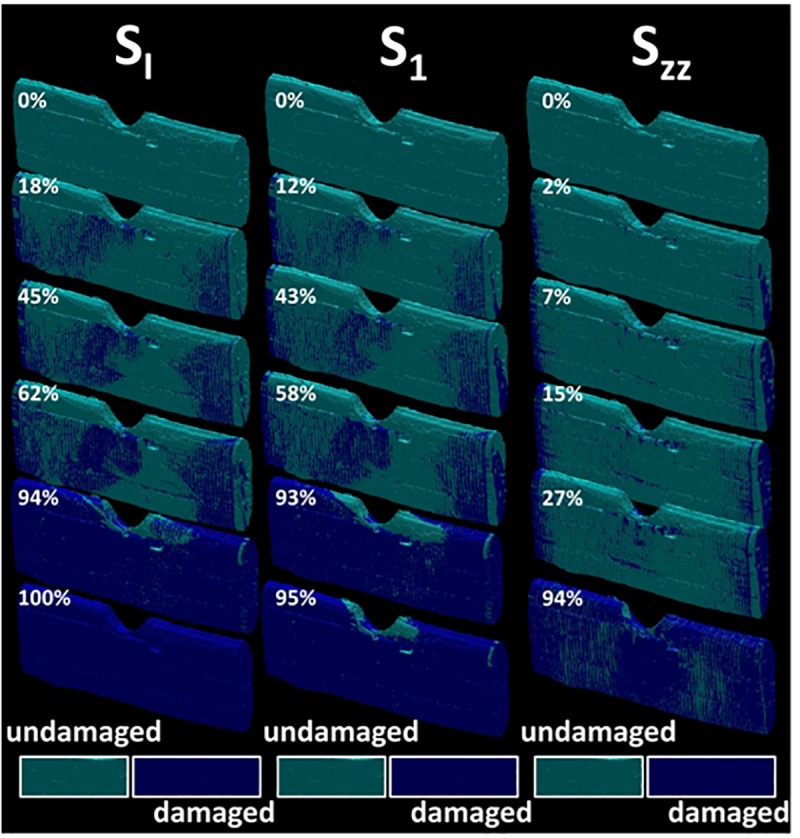
Evolution of the damage ratio as a function of the load level for different damage stress criteria S_I_, S_1_, and S_ZZ_ (U-notch (FXU4). The damage ratio is labeled for each sequence. The snapshots are extracted based on a regular stepping between the initial load step (image on the top) and the displacement at break (image on the bottom).

**FIGURE 10:**
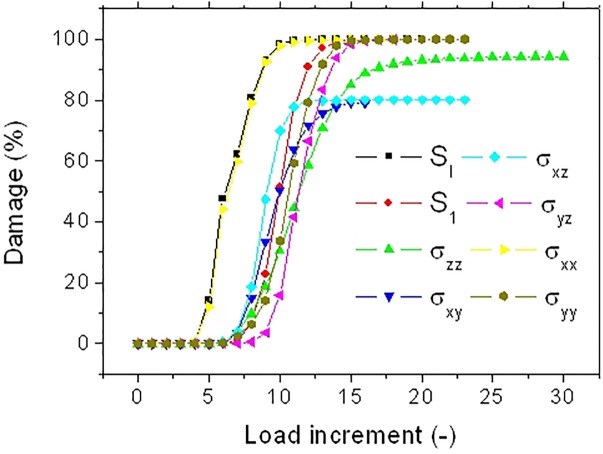
Predicted damage evolution for different stress criteria for a hemp bundle with a U-notch (FXU4).

Using a different coloring for the damaged areas, the localization of damage can be monitored as a function of the load increment as shown in Figure 9. Different damage patterns occur depending on the type of stress criterion. A more localized damage at the end points is depicted for the damage criterion S_ZZ_. Only minor differences between damage scenarios are observed between S_I_ and S_1_ criteria. These scenarios highlight a more diffuse damage in the transverse direction connecting the notch to the fiber ends. A close look at the bundle end reveals that the damage is not exclusively localized at the surface of the bundle but it calls for the contribution of the lumen space. Indeed, even if the loading direction is aligned with the lumen space, the tubular porosity acts as a stress concentrator. As a consequence, damage localization within the core of the bundle occurs and develops at earlier load increments. The damage at the lumen space is common to all damage criteria but with different extents. By summing up all damaged elements divided by the total number of elements that can be possibly damaged during the simulation, the damage ratio can be derived for each load increment. This damage ratio labeled in Figure 9 is plotted against the load increment in Figure 10 for the eight tested damage criteria to derive the damage kinetics. The obtained curves reveal for all damage criteria a three-stage damage kinetics represented by the damage onset, growth and saturation. The damage onset is earlier for the criteria S_I_, σ_XX_. These damage criteria can be considered as more aggressive because the stress state within the bundle rapidly vanishes (Figure 8). Shear stress criteria (σ_XY_, σ_XZ_, σ_YZ_) tend to have a lower saturation regime because the leading deformation mechanism is traction. These stress criteria also tend to produce a smoother damage growth compared to the remaining criteria.

**FIGURE 11:**
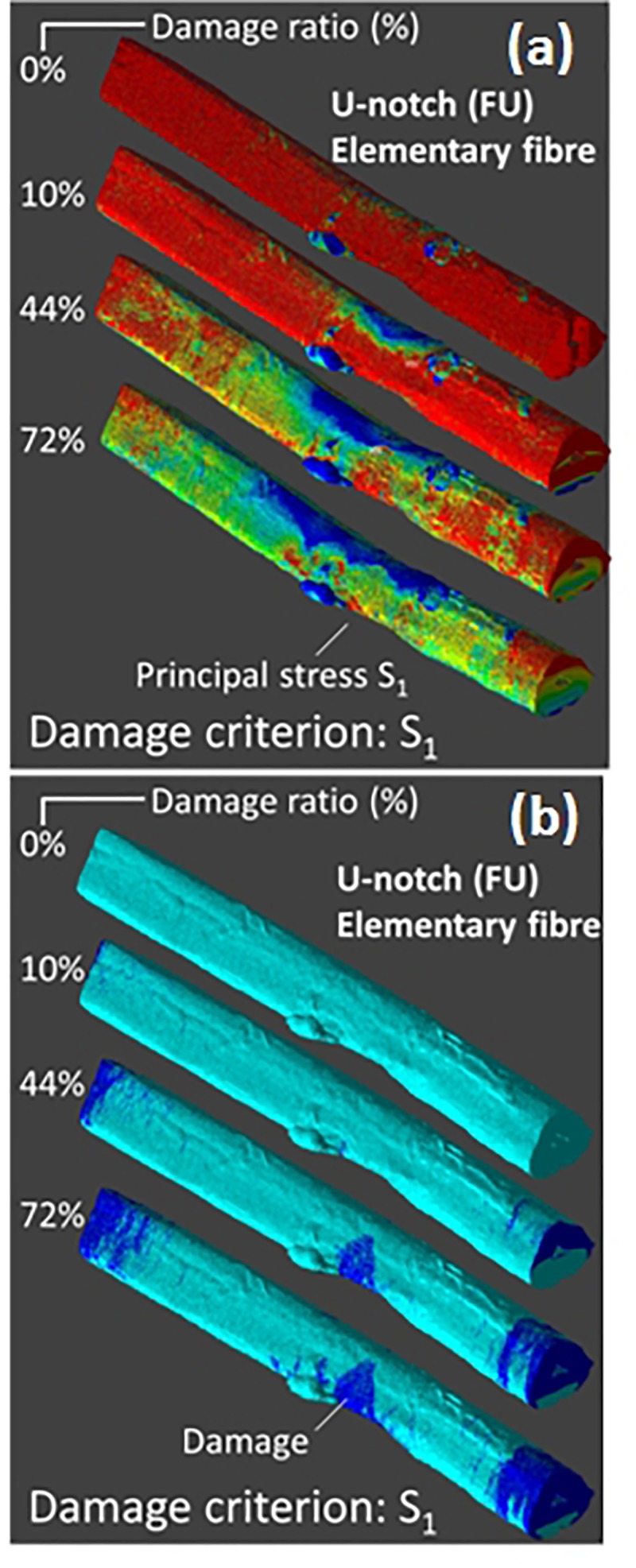
Finite element computation results showing the **(a)** principal stress distribution and **(b)** damage evolutions for the damage criterion S1 under tensile loading by 8% of an elementary hemp fiber with a U-notch (FU). Computations are performed with up to 36.37 × 10^6^ dof.

Figure 11 shows a more contrasted simulation related to the loading of a U-notched bundle. This fiber has a limited lumen space with a porosity level of only 4% a nearly rounded shape (shape factor of 1.28) and a diameter of 44 μm. The computation is performed on an (Region of Interest) ROI containing 13.08 million voxels (0.29 × 10^6^ μm^3^) for a length of 240 μm.

The damage criterion illustrated here is the principal stress component S_1_. For early load increments, a more localized stress drop at mid-length of the fiber is revealed. Examination of the damage patterns shows that two particular regions are concerned: fiber ends and the notch area as in the former case. However, there seems to be no connectivity between these two regions. Moreover, the damage pattern seems to be not fully correlated to the surface view of the stress field. This indicates none negligible contribution of a volume damage.

**FIGURE 12:**
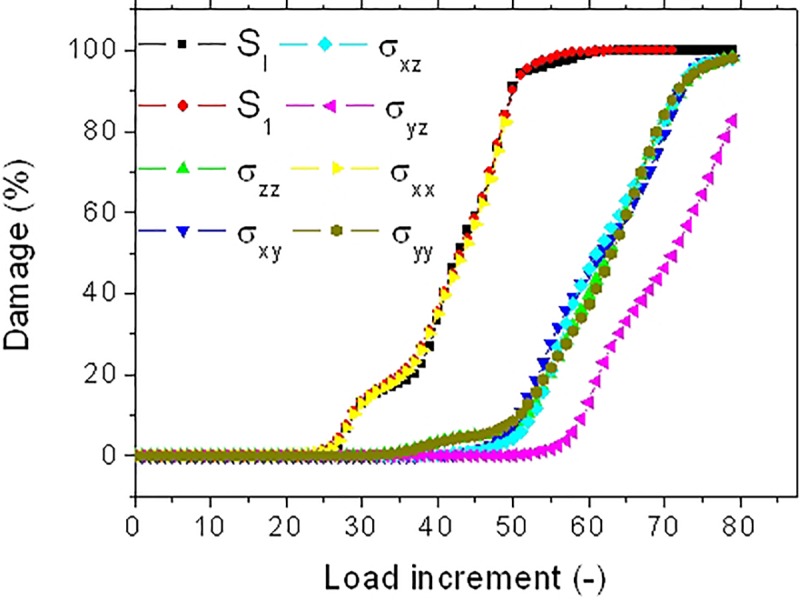
Predicted damage evolution for different stress criteria for an elementary hemp fiber with a U-notch (FU).

Figure 12 depicts the damage kinetics for all tested damage criteria. Although, the three damage regimes are observed, there are marked differences in the damage growth if compared with the damage kinetics of the bundle in Figure 10. There is an evidence of change in the damage rate within the growth stage. This change in damage kinetics is attributed to the transition from a localized to a diffuse damage growth (Figure 11). In addition, noticeable differences between damage criteria can be read. These can be categorized into three main groups. The first category comprises the criteria S_I_, S_1_, and σ_XX_. These criteria trigger an early damage onset, a bimodal damage growth and full damage saturation. The second category allows intermediate damage kinetics. The concerned criteria are a mix of sharing stress components along the loading direction (σ_XZ_, σ_XY_) and normal stress components (σ_Y Y_, σ_ZZ_). For these criteria, the resultant damage kinetics a delayed damage onset, an extended damage tail referring to a slow damage progress followed by a fast damage growth and, finally, late damage saturation. The last category is represented by the shearing stress component normal to the loading direction σ_Y Z_. For this criterion, the damage progress is the most inefficient one because of the low stress levels within the bundle. The damage development occurs with a less evident bimodal growth rate. Saturation should be observed beyond the maximum implemented load increment.

**FIGURE 13:**
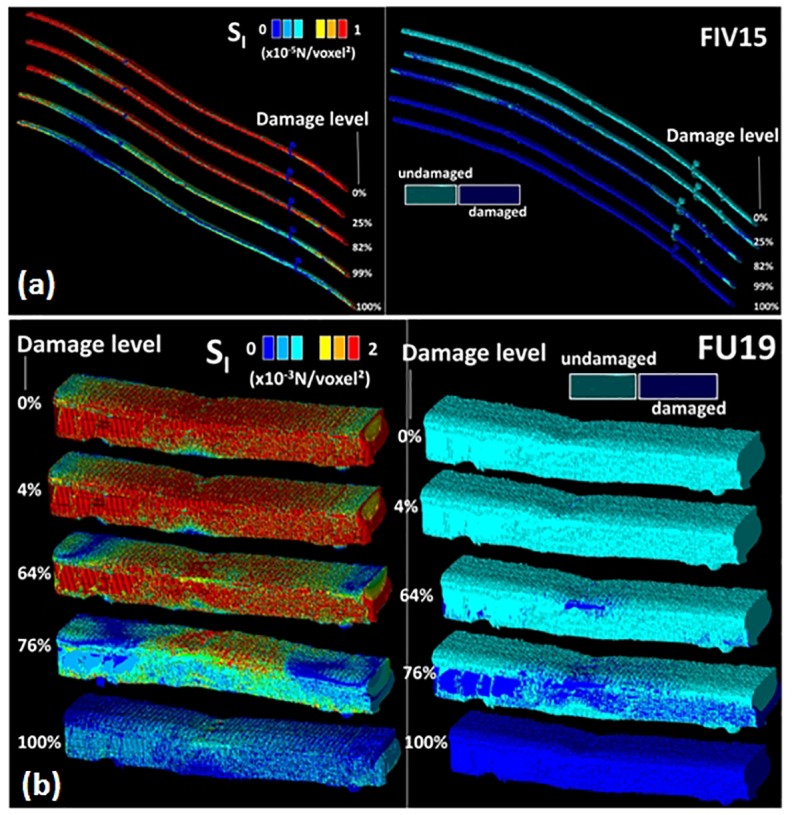
Predicted stress intensity and damage ratio for a hemp elementary fiber with **(a)** a V-notch (FIV15) and **(b)** a U-notch (FU19) using a stress intensity damage criterion.

Figure 13 compares the stress intensity evolution for two contrasted examples of hemp bast fibers, namely, a lengthy elementary fiber with V-notch (FIV15) and a U-notched thicker fiber with no lumen space (FU19). FIV15 is the smallest fiber in lateral dimensions (diameter of 18 μm) and has no lumen space. The ROI contains 2.06 million voxels (45.24 × 10^3^ μm^3^) for a length of more than 580 μm. FU19 is slightly larger with 23 μm in diameter with no lumen and a more elongated shape (the lateral shape factor is 1.78). The computation is performed on a part of the fiber containing 2.92 million voxels (64.23 × 10^3^ μm^3^) for a length slightly of larger than 160 μm.

In the first case (FIV15), a limited influence of the fiber ends on the stress concentration is evident. However, due to the small lateral dimension of the fiber, localization of the stress at different locations in the fiber is observed. Thus, the stress heterogeneity that takes place in the fiber is not exclusively limited to the notched area. This translates the increasing influence of the surface defects that contribute as much as the defect introduced by microdissection. Some of the delaminated parts of the fiber exhibit low stresses. To check if these are the result of damage or weak load transfer, the damage patterns are examined (right side in Figure 13). The damage patterns clearly show that these parts do not play any role during the loading as these are weakly connected to the rest of the fiber. Thus, there is limited load transfer here and most of the influence is attributed to the surface defects. In contrast, the fiber with a U-notch (FU19) promotes a larger contribution of the fiber bulk and a more diffuse damage that connect the end points with the central area containing the notch.

**FIGURE 14:**
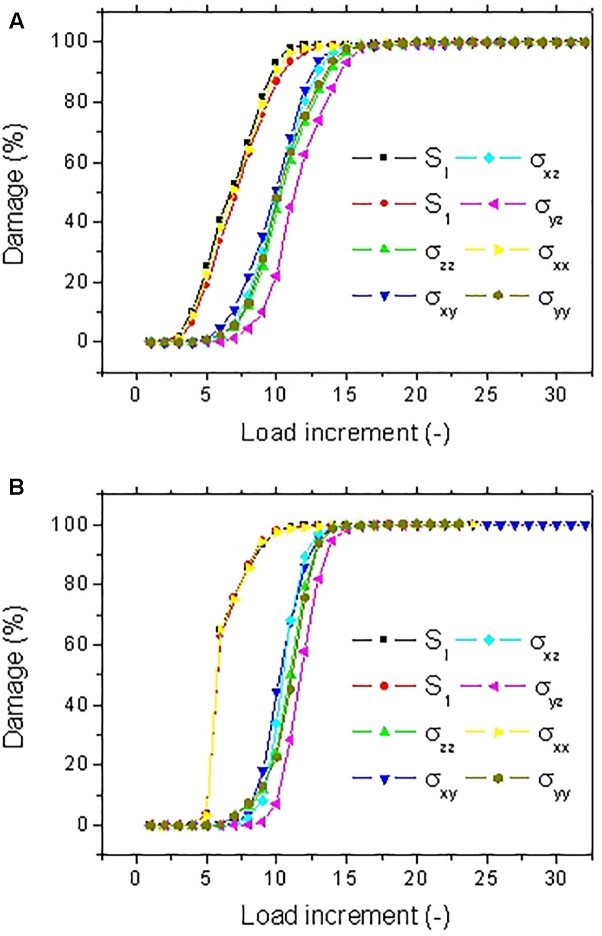
Predicted damage evolution for different stress criteria for a hemp elementary fiber with **(A)** a V-notch (FIV15) and **(B)** a U-notch (FU19).

The consequence of these two contrasted situations on the kinetics of damage growth is illustrated in Figure 14. For the fiber FIV15 (Figure 14A), the observed damage growths share the same trends as the ones related to the behavior of FX04 (Figure 10). For the two situations, the damage growth is monomodal with nearly a constant damage rate. However, among the stress criteria, three allows a more aggressive damage evolution (S_I_, S_1_, and σ_XX_).

A similar ranking of the criteria in terms of damage development is achieved for FU19 (Figure 14B). However, the large damaged areas depicted in Figure 13 at early load increments suggest a faster damage growth. A more abrupt damage growth is evident in Figure 14B especially for the most aggressive criteria (S_I_, S_1_, and σ_XX_).

## Conclusion

The combination of ultra-fine resolution (voxel size of 280 nm) X-ray micro-tomography and FE computation reveals different damage kinetics depending on the hemp bast fiber characteristics and the stress criterion used to lower locally the fiber properties. The common feature for all damage trends is the presence of a three-stage evolution quantified from the analysis of damage ratio as a function of the load increment. The onset, damage growth and saturation are the main identified stages. All these depend on the fiber attributes that include the porosity content, the geometry and dimensions, the surface and bulk defects. It is found that among the stress criteria stress intensity S_I_, principal stress component S_1_ and to a lesser extent normal stress component σ_XX_ trigger severe damage evolution. Analysis of damage patterns suggests that fiber ends, notches and lumen space are the regions of more severe damage sites. In terms of damage rate, the study concludes that the development of a biomodal or a monomodal damage growth is correlated to the presence of localized or diffuse damage. Although the damage model is a first step to partly capture the non-linearity during the tensile loading of the fiber, the conclusions and the validity of the computations strongly rely on the material’s model used. In this study, the hypothesis of a linear elastic isotropic material is a strong hypothesis when compared to the observed non-linear inelastic behavior of the fiber wall material. A future work would clarify the contribution of the inelastic behavior of the fiber wall material from the non-linearity driven by damage development.

## Data Availability

The raw data supporting the conclusions of this manuscript are available to all qualified scientists upon request.

## Author Contributions

SG contributed to the overall management of the work, performed the computations, and contributed in the mechanical testing experiments. He also contributed to the analysis, interpretation of experimental data including testing, X-ray micro-tomography, and finite element computations. He developed the damage based model for bast fibers and contributed substantially to the drafting the article. JB was in charge of the processing of hemp bast fibers. He also contributed in the mechanical set-up for the testing of the fibers, performed the mechanical tests, analyzed the experimental results, contributed in the acquisition of tomograms and contributed to the drafting of the manuscript.

## Conflict of Interest Statement

The authors declare that the research was conducted in the absence of any commercial or financial relationships that could be construed as a potential conflict of interest. The handling Editor is currently editing co-organizing a Research Topic with one of the authors JB, and confirms the absence of any other collaboration.
